# Supplementary material for: Application of observational research methods to real-world studies for rare disease drugs: A scoping review protocol
Source: PLoS One. 2025 Mar 28;20(3):e0304540. doi: 10.1371/journal.pone.0304540 (PMC11952218; doi:10.1371/journal.pone.0304540)
Supplement: S1 Appendix — (DOCX) [file pone.0304540.s001.docx]

**S1 Appendix. Search strategy**

**MEDLINE**

Search conducted on Oct 31, 2023

| 1 | exp Rare Diseases/ | 14098 |
| --- | --- | --- |
| 2 | exp Orphan Drug Production/ | 1483 |
| 3 | ((rare or orphan) adj2 disease*).tw,kf. | 49303 |
| 4 | ((rare or orphan) adj2 drug*).tw,kf. | 2751 |
| 5 | or/1-4 [**rare disease] | 60722 |
| 6 | exp Observational Studies as Topic/ | 9100 |
| 7 | exp Retrospective Studies/ | 1152042 |
| 8 | ((observational adj2 stud*) or research).tw,kf. | 2311724 |
| 9 | or/6-8 [**Observational research] | 3381917 |
| 10 | real-world evidence.mp. | 4876 |
| 11 | real-world data.mp. | 10904 |
| 12 | real-world stud*.mp. | 4475 |
| 13 | or/10-12 [**real-world studies] | 18495 |
| 14 | 9 or 13 | 3392850 |
| 15 | 5 and 14 | 8716 |
| 16 | limit 15 to yr="2018 -Current" | 4645 |

**EMBASE**

Search conducted on Oct 31, 2023

| 1 | exp Rare Diseases/ | 50479 |
| --- | --- | --- |
| 2 | exp Orphan Drug Production/ | 3910 |
| 3 | ((rare or orphan) adj2 disease*).tw,kf. | 80088 |
| 4 | ((rare or orphan) adj2 drug*).tw,kf. | 5308 |
| 5 | or/1-4 [**rare disease] | 112389 |
| 6 | exp Observational Studies as Topic/ | 342056 |
| 7 | exp Retrospective Studies/ | 1512126 |
| 8 | ((observational adj2 stud*) or research).tw,kf. | 3041887 |
| 9 | or/6-8 [**Observational research] | 4490980 |
| 10 | real-world evidence.mp. | 9260 |
| 11 | real-world data.mp. | 21416 |
| 12 | real-world stud*.mp. | 8584 |
| 13 | or/10-12 [**real-world studies] | 36056 |
| 14 | 9 or 13 | 4508418 |
| 15 | 5 and 14 | 16656 |
| 16 | limit 15 to yr="2018 -Current" | 9590 |
