# Supplementary material for: Application of observational research methods to real-world studies for rare disease drugs: A scoping review protocol
Source: PLoS One. 2025 Mar 28;20(3):e0304540. doi: 10.1371/journal.pone.0304540 (PMC11952218; doi:10.1371/journal.pone.0304540)
Supplement: S2 Appendix — (DOCX) [file pone.0304540.s002.docx]

**S2 Appendix. Data extraction form**

| **General article information** | | |
| --- | --- | --- |
| Title |  | |
| First Authors |  | |
| Journal |  | |
| Year |  | |
| DOI |  | |
| Country(s) |  | |
| Continent |  | |
| Funding source(s) |  | |
|  | | |
| **Study overview** | | |
| Study design |  | |
| Rare disease being studied |  | |
| Rare disease intervention being used |  | |
| Primary study aim/objective |  | |
|  | | |
| **Data sources** | | |
| Data sources used |  | |
| Healthcare setting |  | |
|  | | |
| **Methodology** | | |
| Observational research method(s) used |  | |
| Authors’ rationale for use of the method(s) |  | |
|  |  | |
| **Outcomes** | | |
| Outcomes being measured |  | |
| How are the outcomes defined and measured |  | |
|  | | |
| Results | |  |
| Summary of key findings | |  |
| Conclusions | |  |
