# Supplementary material for: Application of observational research methods to real-world studies for rare disease drugs: A scoping review protocol
Source: PLoS One. 2025 Mar 28;20(3):e0304540. doi: 10.1371/journal.pone.0304540 (PMC11952218; doi:10.1371/journal.pone.0304540)
Supplement: Attachment — Submitted filename: Response to Reviewers.docx [file pone.0304540.s006.docx]

**Responses to Editor Suggestions and Comments**

Reviewer #1: In the study protocol “Application of observational research methods to real-world studies for rare disease
treatments: a scoping review protocol,” the authors outline a methodology to identify observational research methods used over the past 5 years and evaluate their application. This work is relevant to those closely related to their field.

Minor revision suggestions:
• In the introduction, please place references 1 – 4 with the respective piece of information rather than all together at the end.

Response: Addressed. Thank you for your suggestion.

• Under exclusion criteria the authors state English as a language restriction. A scoping review should not technically have a language restriction. If this is appropriate in this case, it should be justified.

Response: Language restriction will be removed. Thank you for your suggestion.

• Under exclusion criteria the authors mention that case reports and series will be excluded, however these are often also observational in nature; please clarify.

Response: During a preliminary search we found that case series and reports were more descriptive vs. quantitative, and many studies did not detail methods to employed in the study. To ensure feasibility given the broad nature of the search we excluded them.

• Consider adding the use of a medical librarian to assist in setting up key words and concepts.

Response: A librarian was consulted during the preliminary search which aided in the construction of the search terms.

Reviewer #2: Title: Looks interesting and identifies the report as a scoping review protocol
Short title: Observational research methods for rare disease treatments (suggestion)

Response: Thank you for your suggestion this is much easier to read.

Abstract:
7000 different conditions looks like a huge number of diseases, are the authors looking at any specific diseases (it might be good to keep the diseases to one condition or specialty) for better results (suggestion).

Response: There are a considerable number of conditions that qualify as a rare disease. As such we will be using the Health Canada definition, which is also aligned with the European Commission definition. The EC has a website which notes all rare conditions so diseases will be cross-checked on there.

Can the authors change their search duration till 2024?

Response: We will update the wording to suggest 5 years from the time of the search execution to ensure relevancy and timeliness.

Languages?

Response: Language restriction will be removed. Thank you for your suggestion.

Will they consider implementation studies?

Response: Thank you for your suggestion but based on the preliminary search, implementation studies would be out of scope for this review.

MEDLINE and EMBASE looks very narrow for this research question? Please consider adding subject specific databases, if none exist – please mention

Response: Based on consultation with a librarian these databases were deemed most appropriate. To ensure feasibility and scope they were selected.

Some terms like – observational research methods, rare disease would be helpful for readers and standardizing the terminology.

Response: Thank you for your suggestion.

Competing interests:
Looks like MT has some conflicts of interests reported in a paper, this can be mentioned here in this paper as well?
“M. T. received financial support from the Canadian Agency for Drugs and Technologies in Health. M. T., T. A., and K. N. H. report a relationship with Canadian Agency for Drugs and Technologies in Health that includes consulting or advisory.”
(ref: Development of a Canadian Guidance for reporting real-world evidence for regulatory and health-technology assessment (HTA) decision-making., https://doi.org/10.1016/j.jclinepi.2024.111545. Journal pre proof)

Response: Thank you for your suggestion. We will note MT conflict of interest in the appropriate section.

If there are concerns with conventional RCTs it might be helpful to look at concerns related to efficacy, safety evidence too?

Response: Thank you for your suggestion. However, the scope of this review will be limited to evidence from observational studies.

Can they address concerns with real world studies as well? They aren’t planning to look at RCTs.

Introduction:
Nice introduction.

Response: Thank you for your kind words.

It might be helpful to mention how observational studies may contribute to effectiveness in real world drug (line 97,98)

Response: Adjusted the wording to incorporate. Thank you for your suggestion.

Can a reference be added for statement 107,108 please?

Response: Addressed. Thank you for your suggestion.

Imperative sounds too strong – can this very be changed to ‘might be helpful’. If its imperative, might as well perform RCTs?

Response: Adjusted. Thank you for your suggestion.

Rare diseases – looks very broad, can the authors consider narrowing the participants?
Can they also explain and add some detail on the existing research methods for better understanding. And expand on the ‘information sources’ please. Add a note on grey literature search as well. Can they also add a note on how they will conduct their supplementary search (line 151-153) especially in relation to relevant literature searches.

Response: Thank you for your suggestion. Given the broad nature of this review a supplementary search of titles and abstract of references from the included articles list will be conducted.

Data management: How they will manage data using Excel is unclear, if they could explain which data will be managed it might be helpful.

Response: Thank you for your suggestion.

The inclusion criteria need to be bit more robust and detailed to prevent any future concerns during the screening phase- hope the authors will consider the suggestion to make the criteria a bit more detailed.

Response: Thank you for your suggestion.

Regarding missing data – line 219 to 220, is there are a time frame the authors will wait while they contact the study authors, any time frames for sending reminders?

Response: Adjusted accordingly. Thank you for your suggestion.

Will they use Covidence for data extraction as well? Might be good to mention it here.

Response: Thank you for your suggestion. Excel will be used for data extraction.

Data analysis and presentation:
It might be good to mention the type of quantitative and qualitative analysis that will be performed please?

Response: Thank you for your suggestion. Line 225-231 notes the quantitively analysis that will be taken. We’ll most use summary statistics to provide the frequency of the extracted data. Qualitative analysis will be descriptive in nature.

Inclusion criteria: please explain the concept in detail to prevent ambiguity, like conditions or please consider adding an appendix

Response: Thank you for your suggestion.

Context: the term ‘health administrative data’ sounds confusing please explain.

Response: Thank you for your suggestion. We realized this is restrictive and adjusted the language accordingly.

I am not sure how the authors are planning to search unpublished studies? Please expand the types of sources.

Response: Thank you for your suggestion. We’re going to remove the unpublished studies as it’s out of scope and not feasible.

Others: Please consider updating the PRISMA ScR checklist based on the revision 1 protocol.

Response: Thank you for your suggestion.

A few questions:
Not sure how they might be able to generate evidence on drug effectiveness or safety?
It might be good to explain real world effectiveness methods
Rare disease drugs and evaluation.

Response: Thank you for your suggestion.
